# Deep-Sea Biological Detection Method Based on Lightweight YOLOv5n

**DOI:** 10.3390/s23208600

**Published:** 2023-10-20

**Authors:** Zhongjun Ding, Chen Liu, Dewei Li, Guangrui Yi

**Affiliations:** 1 National Deep Sea Center, Qingdao 266237, China; dzj@ndsc.org.cn (Z.D.); ldw@ndsc.org.cn (D.L.); 201701091028@sdust.edu.cn (G.Y.); 2 College of Electrical Engineering and Automation, Shandong University of Science and Technology, Qingdao 266590, China

**Keywords:** deep-sea biological detection, image enhancement, YOLOv5n, transfer learning, knowledge distillation, lightweight

## Abstract

Deep-sea biological detection is essential for deep-sea resource research and conservation. However, due to the poor image quality and insufficient image samples in the complex deep-sea imaging environment, resulting in poor detection results. Furthermore, most existing detection models accomplish high precision at the expense of increased complexity, and leading cannot be well deployed in the deep-sea environment. To alleviate these problems, a detection method for deep-sea organisms based on lightweight YOLOv5n is proposed. First, a lightweight YOLOv5n is created. The proposed image enhancement method based on global and local contrast fusion (GLCF) is introduced into the input layer of YOLOv5n to address the problem of color deviation and low contrast in the image. At the same time, a Bottleneck based on the Ghost module and simAM (GS-Bottleneck) is developed to achieve a lightweight model while ensuring sure detection performance. Second, a transfer learning strategy combined with knowledge distillation (TLKD) is designed, which can reduce the dependence of the model on the amount of data and improve the generalization ability to enhance detection accuracy. Experimental results on the deep-sea biological dataset show that the proposed method achieves good detection accuracy and speed, outperforming existing methods.

## 1. Introduction

Deep-sea biological activities are crucial in shaping the marine ecosystem and regulating global climate, driving the oceanic element cycling from short to long timescales [1,2]. Underwater target detection is an efficient way to study and conserve deep-sea biological resources. However, the imaging environment in the deep sea is highly complex, leading to significant challenges in capturing high-quality deep-sea optical images using underwater cameras. These images often exhibit color shifts, low contrast, and limited sample size, which ultimately hamper the accuracy of deep-sea biological object detection.

In recent years, extensive research has been conducted in the field of underwater biological detection. The current underwater biological detection algorithms can be divided into two types of methods: traditional machine learning-based and deep learning-based. Traditional machine learning-based methods extract object features and feed them to a classifier for detection. Zhang et al. [3] first enhanced underwater images, removed target features through image segmentation and feature extraction, and used support vector machines for detection. Chen et al. [4] employed the speed-up robust features algorithm to extract target features, represent features using the bag of words method, and use random forests for detection. However, difficulties in feature extraction and poor robustness are encountered due to the complexity of underwater environments and the need to manually design various window sizes and feature extractors in traditional machine learning-based algorithms.

Deep learning-based algorithms can be classified into two categories. The first category is two-stage algorithms, in which a region proposal network is employed to generate regions of interest. These selected regions are subsequently classified using the network. Exemplary algorithms within this category comprise R-CNN [5] and Faster R-CNN [6]. Tan et al. [7] designed an image preprocessing method of the MSRCR and median filtering, improved the neck network using attention mechanisms, and achieved high accuracy. Although this class of methods achieves high accuracy, they tend to have slower detection speeds. Another category consists of one-stage algorithms, exemplified by YOLO [8]. These algorithms treat object detection’s localization and classification tasks as regression questions, leading to faster detection speeds. Hao et al. [9] applied the automatic color enhancement algorithm to enhance the color and brightness of MSRCR-processed images, replacing residual modules in the YOLOv3 feature extraction network with dense blocks to improve accuracy. Wageeh Y et al. [10] used the MSRCR to enhance the quality of underwater images. They combined the YOLO algorithm with the optical flow to achieve fish detection and tracking. Zhang et al. [11] proposed an underwater target detection model based on channel attention and feature fusion. They also designed a data augmentation method based on concatenation and fusion to effectively reduce the occurrence of false positives and false negatives in the model.

The above research on deep learning-based algorithms has progressed. However, there are three primary challenges for deep-sea biological detection: (1) those due to the deep-sea biological images acquired in the low-light deep-sea environment, in addition to color deviation, and the image having low contrast, overexposure, and underexposure, which affect detection accuracy; (2) the acquisition of deep-sea biological images is expensive, leading to insufficient image samples and limited model generalization ability; and (3) most current models for underwater biological detection improve detection accuracy at the cost of complexity, resulting in high model complexity that may not be more conducive for deployment in small deep-sea detection devices. 

To solve the abovementioned challenges, a deep-sea biological detection method based on the lightweight YOLOv5n is proposed. The main contributions of this paper are:

(1) We propose an image enhancement method based on global and local contrast fusion for low-quality deep-sea biological images. Gamma correction, CLAHE, and PCA are applied to improve the contrast of the image after correcting the image color based on the grayscale world.

(2) A lightweight Bottleneck module is designed, which combines the advantages of cheap operation of the Ghost module and the advantages of simAM to focus on important biological features and realizes the lightweight of the model under a certain detection performance. 

(3) We propose a transfer learning strategy combined with knowledge distillation for insufficient deep-sea biological image samples. The strategy migrates similar characteristics of shallow-sea organisms into the model and further improves the generalization ability of the model by knowledge distillation to mitigate the effect of insufficient data on the detection accuracy without increasing the model complexity.

The rest of this paper is organized as follows: The methodology of the proposed method is presented in Section 2. The effectiveness of the proposed method is demonstrated in Section 3. The conclusions are drawn in Section 4.

## 2. Methods

### 2.1. Overall Structure of the Method

**Figure 1:**
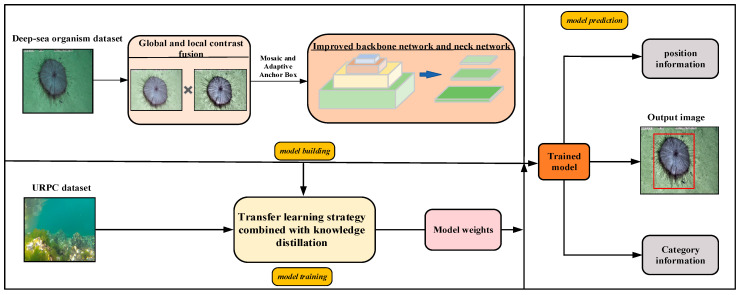
Overall framework of the proposed method.

This method mainly consists of constructing a lightweight YOLOv5n detection model and a transfer learning strategy combined with knowledge distillation. As shown in Figure 1, in terms of model construction, the designed image enhancement method based on global and local fusion is added to the model to enhance deep-sea biological images. Secondly, to build a lightweight model, a GS-Bottleneck is designed based on the Ghost module and the simAM, and it is used to replace the neck network in the original backbone network and neck network to obtain the improved backbone network and neck network. In terms of model training, the models are trained on the deep-sea organism dataset and shallow-sea biological dataset using a transfer learning strategy combined with knowledge distillation. After several iterations, the weight parameters are assigned to the model. The trained model can detect the position information and class information of the organisms in the image.

### 2.2. Lightweight YOLOv5n Detection Model

#### 2.2.1. Overall Framework of the Model

**Figure 2:**
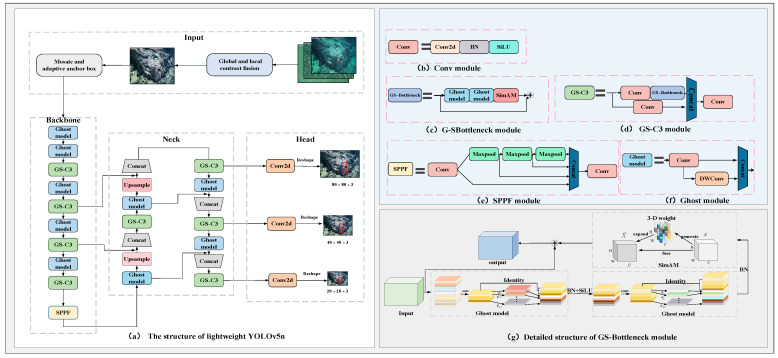
Overall structure and basic modules of lightweight YOLOv5n.

In order to decrease the complexity of the model and solve the impact of low-quality images on the model’s accuracy, a lightweight YOLOv5n model is designed based on the YOLOv5n model [12]. As shown in Figure 2, in the input layer of the model, the designed image enhancement method based on global and local contrast fusion is introduced to correct the image’s color, improve the image contrast, and facilitate the feature extraction of the model. In the backbone network and neck network of the model, the Bottleneck module is substituted with GS-Bottleneck, which is based on the Ghost module and simAM. This substitution significantly decreases the complexity of the model while possessing reliable detection performance. The GS-Bottleneck module substitutes the Bottleneck module in the original C3, leading to the creation of GS-C3. Finally, the Ghost module is used to replace the rest of the ordinary convolutions in the backbone network and the neck network. 

#### 2.2.2. Deep-Sea Biological Image Enhancement Method Based on Global and Local Contrast Fusion

**Figure 3:**
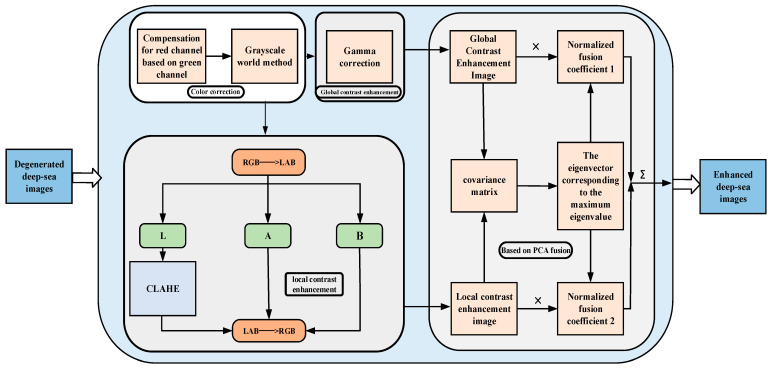
Technology roadmap of deep-sea biological image enhancement method based on global and local contrast fusion.

The natural light in the deep-sea environment has disappeared, and manned submersibles must use artificial light to take images of deep-sea organisms. In addition to the color shift problem, the main challenges of images under such imaging conditions are local overexposure, surrounding underexposure, and low contrast. Such degraded deep-sea images can affect the accuracy of object detection. Therefore, this section proposes a deep-sea biological image enhancement method based on global and local contrast fusion to solve the above problems. As shown in Figure 3, first, a grayscale world-based color correction method is designed, which pre-compensates the r-channel with the g-channel and removes the image’s color cast using the grayscale world method. Second, gamma correction is applied to the color-corrected image to obtain a globally contrast-enhanced version. At the same time, the color-corrected image is converted to LAB space, and CLAHE is applied to the L channel to obtain a locally contrast-enhanced version. Finally, to integrate the complementary advantages between the two versions, the PCA algorithm is used to fuse the two versions to obtain the enhanced images. The fusion strategy maintains the enhanced image’s advantages of global contrast, local contrast, and natural color. In addition, the algorithm has low complexity and fast computation speed to meet the real-time enhancement requirements. 

When light propagates in seawater, the red color decays the fastest, resulting in a blue-green bias in the image. Moreover, it has been proven in the literature [13] that color domain shift causes the loss of model accuracy. This paper uses a color correction method based on the gray world to correct the color cast. Considering the particularity of the propagation and attenuation of visible light, the pixel mean of the red channel of the deep-sea image is relatively small, and the gray world assumes that the pixel mean of different channels in the scene should be equal. Therefore, when using the gray world method for color restoration, the red channel is excessively increased, resulting in color artifacts in the enhanced deep-sea image. To solve the problem, firstly, the red channel is pre-compensated through Equation (2):

(1) $$I_{rc}(x)=I_{r}(x)+\alpha \cdot \left(\bar{I_{g}}-\bar{I_{r}}\right)\cdot \left(1-I_{r}(x)\right)\cdot I_{g}(x)$$

where $I_{rc\;}(x)$ and $I_{r\;}(x)$ are the red channel values after and before compensation, ${\bar{I}}_{g\;}$ and ${\bar{I}}_{r\;}$ are the mean values of the blue and red channels, respectively. α is a constant parameter. After the red channel is compensated, the color cast of the image is corrected using the gray world method [14], which adjusts the color of each pixel by calculating the average gray value of the image (the average of all pixel values), so that the gray value of each pixel matches the average gray value of the whole image, so as to realize the equalization and naturalization of the image color. Then, gamma correction is applied to the color-corrected image to obtain a global contrast-enhanced image. Gamma correction performs a nonlinear transformation of the brightness value of the image through Equation (2) to adjust the overall contrast of the image.

(2) $$I_{out\;}(m,n,c)=vI_{in}^{\gamma }(m,n,c)$$

where $I_{in}(m,n,c)$ is the input image intensity and $I_{out\;}(m,n,c)$ is the output image intensity. *m* and *n* represent pixel coordinates, while *c* represents the color channel index. *γ* and *v* are the parameters used to adjust the shape of the gamma function.

In order to better enhance the local detail features in the image, and considering that gamma correction has a good enhancement effect on underexposed images, the CLAHE algorithm has obvious advantages in overexposed image enhancement [15]. Hence, this paper uses CLAHE for local contrast enhancement of images. CLAHE [16] is an adaptive histogram equalization method that divides the image into small blocks and limits the number of pixels in each gray level to make the gray level distribution in each small block more uniform, and finally uses the interpolation method to obtain the contrast-enhanced image. Therefore, we transfer the color-corrected images from the RGB space to the LAB space and use the CLAHE method in the L channel to reduce the computational effort and avoid color distortion while improving the local contrast of the image.

The global and local contrast enhancement of the integrated enhanced image can highlight the details and texture features of the image while maintaining the overall contrast of the image. Therefore, an image fusion method based on PCA [17] is designed in this section, and the characteristics of PCA are used to efficiently fuse the two images to achieve complementary advantage features. The core of the method is that PCA can retain the main information in the original data, the covariance matrix of the data is obtained from the source image, and the eigenvector corresponding to the largest eigenvalue of the covariance matrix is used to determine the weighting coefficient in the image fusion algorithm. Finally, the fused image is obtained through the coefficient and the source image. The fusion method can quickly retain the information of both images to achieve complementary advantages. For two images, *G*_1_ and *G*_2_, each image is treated as an n-dimensional vector denoted by *P_o_*, *o* = 1, 2, and the image fusion process is as follows:

(1) Compute the covariance matrix *W* of *Po*:

(3) $$W=\left[\begin{array}{l} \sigma_{11}^{2}\\ \sigma_{21}^{2} \end{array}\right.\left.\begin{array}{l} \sigma_{12}^{2}\\ \sigma_{22}^{2} \end{array}\right]$$

where $\sigma_{ij}^{2}$ is the variance of the image.

(2) Compute the eigenvalues (*K*_1_, *K*_2_) and eigenvectors (*ξ*_1_, *ξ*_2_) of the covariance matrix *W*.

(3) Choose the large eigenvalue λ:

(4) $$\lambda =\arg\max\left(\lambda_{1},\lambda_{2}\right)$$

(4) Calculate the weight coefficient according to the eigenvector corresponding to the larger eigenvalue and obtain the fused image *R*:

(5) $$c_{1}=\frac{\xi_{1}}{\xi_{1}+\xi_{2}}\;\mathrm{and}\;c_{2}=\frac{\xi_{2}}{\xi_{1}+\xi_{2}}$$

(6) $$R=c_{1}G_{1}+c_{2}G_{2}$$

#### 2.2.3. GS-Bottleneck

To reduce the model complexity while maintaining a certain level of feature extraction ability, the Bottleneck module in YOLOv5n is improved by using the Ghost module and simAM. This modification resulted in the GS-Bottleneck, as shown in Figure 2g. In the GS-Bottleneck, the ordinary convolution module is substituted with the Ghost module. The Ghost module is known for reducing the number of parameters and calculations, effectively decreasing the model complexity. Although the Ghost module reduces model complexity, it may also result in a decrease in feature extraction ability compared to the original Bottleneck module. This trade-off is a consideration when using the Ghost module. Therefore, the simAM is concatenated after two Ghost modules to focus on important biological features so as to decrease the impact of model accuracy reduction caused by the Ghost modules to a certain extent without increasing the number of parameters.

The Ghost module [18] is a lightweight feature extraction module that makes full use of the redundant characteristics of feature maps generated by ordinary convolution to reduce the number of parameters and calculations. The essence is to decompose one convolutional multiplication into the sum of two convolutional multiplications, thus reducing the number of parameters. The Ghost module first obtains a small number of intrinsic feature maps using one 1 × 1 ordinary convolution. Secondly, cross-feature point feature extraction is performed using depthwise separable convolutions to generate the corresponding additional feature maps. Finally, the obtained two kinds are concatenated to obtain the output feature map. Assuming that the input feature map size is *h*_1_ × *w*_1_ × *c*, the output feature map size is *h*_2_ × *w*_2_ × *n*, the convolution kernel size is *k × k*, and the step size is *s*, the *FLOPs* of the conventional convolution are as follows:

(7) $$FLOPs(a)=n\times h_{2}\times w_{2}\times c\times k\times k$$

The *FLOPs* of the Ghost module are:

(8) $$FLOPs(b)=\frac{n}{s}\times h_{2}\times w_{2}\times c\times k\times k+(s-1)\times \frac{n}{s}\times h_{2}\times w_{2}\times k\times k$$

The ratio of the two is:

(9) $$\frac{FLOPs(a)}{FLOPs(b)}=\frac{n\times c}{\frac{n}{s}\times c\times (s-1)\times \frac{n}{s}}=\frac{s\times c}{c+s-1}\approx s$$

The ratio of the two shows that the computational complexity of the Ghost module is only 1/s times that of conventional convolution. As a result, by replacing the conventional convolution with the Ghost module, we can achieve an equivalent number of feature maps while reducing the computational load and the number of parameters in the model. 

**Figure 4:**
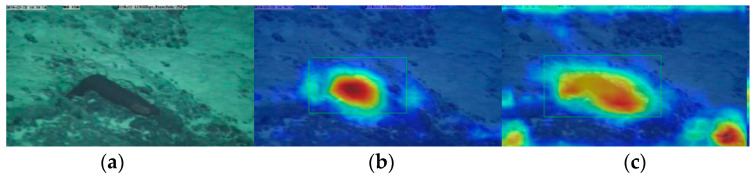
Visualization of heatmap: (**a**) input; (**b**) heatmap before adding simAM; (**c**) heatmap after adding simAM.

The simAM [19] derives three dimension values for the feature map based on brain theory. This allows the model to focus more on the deep feature spatial information of deep-sea organisms, considering the spatial and channel dimensions. Importantly, this attention mechanism achieves this without the need for additional parameters. Specifically, it is believed in neuroscience that neurons with more information have unique firing patterns and inhibit the activity of other neurons, so these neurons can be found by calculating the linear separability between a target neuron and other neurons. The activation heat map of the network was generated using the Grad-CAM [20] algorithm, as shown in Figure 4. The Grad-CAM algorithm can clearly show the area of concern of the network. The figure shows that the model pays more attention to deep-sea organisms after adding simAM. In short, simAM is a lightweight, simple, and effective module. After adding it to the two Ghost modules, it can help the model to screen important deep-sea biological features and make up for the lost accuracy caused by the Ghost module to some extent.

### 2.3. Transfer Learning Strategy Combined with Knowledge Distillation

**Figure 5:**
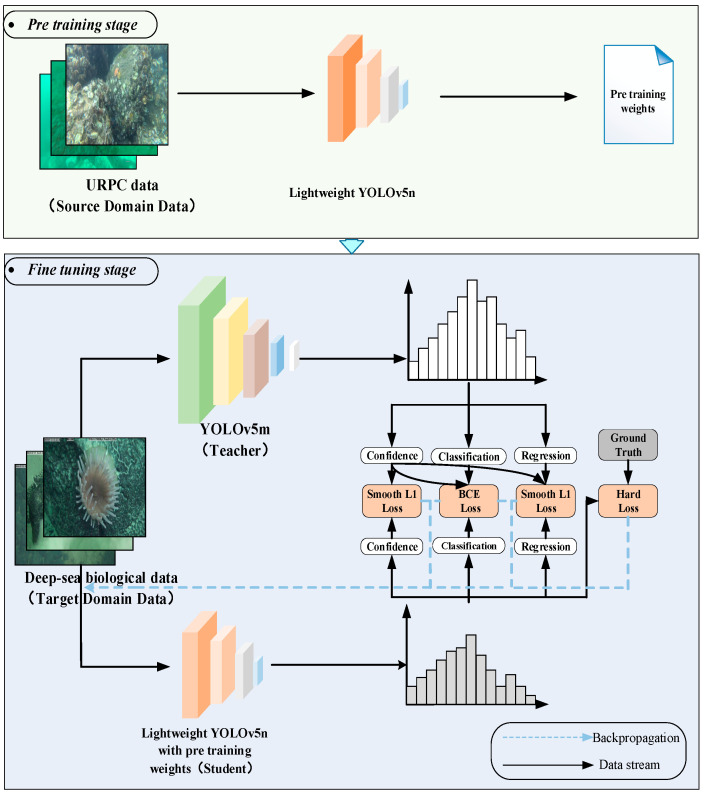
Technology roadmap of transfer learning strategy combined with knowledge distillation.

Deep-sea biological images need to be acquired using manned submersibles or other deep-sea detection equipment. Using this equipment is costly, so the number of deep-sea biological image samples obtained is limited. For the problem of small sample data leading to insufficient generalization ability and low detection accuracy of the model, this section proposes a transfer learning strategy combined with knowledge distillation. This method is based on the framework of transfer learning. As shown in Figure 5, during the pretraining stage, the lightweight YOLOv5n undergoes pretraining using the shallow-sea biological dataset (source domain dataset). This allows the model to learn similar characteristics between shallow-sea and deep-sea biological images. The pretraining weights obtained are then used as initialization parameters for the model in the subsequent fine-tuning stage, thereby improving its performance on the target domain dataset. During the fine-tuning stage, the deep-sea biological dataset (target domain dataset) and a designed knowledge distillation method are utilized to train the model with the pretraining weights. This further optimizes the model’s generalization performance for deep-sea biological detection tasks. YOLOv5n is the least complex version of YOLOv5, so we choose YOLOv5m as the teacher model. In addition, in order to reduce the risk of overfitting of the teacher model, we employ the training technique of early stopping when training the teacher model.

Transfer learning helps to train a reliable decision function in the target domain by transferring auxiliary information source knowledge to solve the learning problem when the sample data in the target domain is unlabeled or has a small number of labeled samples. At the same time, transfer learning releases the restriction of traditional machine learning methods that training data and test data obey the same probability distribution and only require a specific similarity relationship between the source domain and the target domain [21]. In this paper, the URPC dataset is selected as the source data, the lightweight YOLOv5n is pretrained, and its pretrained model weights are retained as the initial weights of the model in the fine-tuning stage.

In order to further improve the generalization ability of the model and increase the detection accuracy of the model on the deep-sea biological dataset with insufficient data, knowledge distillation [22] is introduced into the fine-tuning stage of transfer learning. When retraining on the deep-sea biological image datasets (target domain), the teacher network is used to assist the lightweight YOLOv5n training. One fact is that more complex models often have strong generalization ability, so complex teacher models can achieve higher detection accuracy in deep-sea biological detection tasks. Knowledge distillation is an effective method to improve the model’s generalization ability. It can transfer the knowledge learned by the complex and good performance of the teacher network to the low complexity and poor performance of the student network so that the student network can obtain the accuracy and generalization ability close to the teacher network. In short, in knowledge distillation used in this paper, the knowledge from the teacher model serves as a form of regularization and guides the student model toward better generalization. The loss function of the student network in the object detection task is:

(10) $$L_{}=S_{box}^{}\left(b_{i}^{gt},{\hat{b}}_{i}\right)+S_{cls}^{}\left(p_{i}^{gt},{\hat{p}}_{i}\right)+S_{cof}^{}\left(c_{i}^{gt},{\hat{c}}_{i}\right)$$

where $b_{i}^{gt}$, $p_{i}^{gt}$, and $c_{i}^{gt}$ are the labeled bounding box coordinate value, labeled class probability value, and labeled confidence value, respectively. ${\hat{b}}_{i}$, ${\hat{p}}_{i}$, and ${\hat{c}}_{i}$ are the bounding box coordinate values, class probability values, and confidence values predicted by the object detection model, respectively.

The knowledge distillation based on label knowledge [23] is a commonly used knowledge distillation method. In the process of using this distillation method, there is a problem in that the student network learns a large number of bounding box knowledge predicted by the teacher network in the background area, which affects the bounding box coordinate regression training of the student network. In response to this problem, inspired by the literature [24], this paper redesigns the loss function of the student network. The sigmoid function processes the confidence of the output of the teacher network. At this time, the little confidence tends to 0 after sigmoid function processing, and the student network learns less from the output of the teacher network. On the contrary, when the confidence of the deep-sea biological target predicted by the teacher network is high, the prediction results of the teacher network have a strong guiding effect on the student network. Then, the student network learns the bounding box coordinates and classification probabilities predicted by the teacher network. The confidence loss for the student network is as follows:

(11) $$S_{cof}^{}\left(c_{i}^{gt},{\hat{c}}_{i},{\hat{c}}_{i}^{T}\right)=S_{cof}\left(c_{i}^{gt},{\hat{c}}_{i}\right)+Sigmoid\left({\hat{c}}_{i}{}^{T}\right)\cdot \eta \cdot S_{cof}^{soft}\left({\hat{c}}_{i}^{T},{\hat{c}}_{i}\right)$$

The bounding box regression loss for the student network is as follows:

(12) $$S_{box}^{}\left(b_{i}^{gt},{\hat{b}}_{i},{\hat{b}}_{i}^{T},{\hat{c}}_{i}^{T}\right)=S_{box}\left(b_{i}^{gt},{\hat{b}}_{i}\right)+Sigmoid\left({\hat{c}}_{i}{}^{T}\right)\cdot \eta \cdot S_{box}^{soft}\left({\hat{b}}_{i}^{T},{\hat{b}}_{i}\right)$$

where ${\hat{c}}_{i}{}^{T}$ is the confidence of the teacher network prediction, ${\hat{b}}_{i}^{T}$ is the bounding box coordinate value of the teacher network prediction, $S_{conf}^{soft}$ and $S_{box}^{soft}$ are the mean squared error loss function, and η is the equilibrium factor. The classification loss for the student network is as follows:

(13) Sclspigt,p^i,p^iT,c^iT=Sclspigt,p^i+Sigmoidc^iT⋅c^iT⋅η⋅Sclssoftsoftmax(p^iTT),softmax(p^iT)

where ${\hat{p}}_{i}^{T}$ is the class probability predicted by the teacher, $S_{cls}^{soft}$ is the cross-entropy loss function, and *T* is the temperature at the time of distillation. Combined with the above losses, the loss function for the student network can be expressed as follows:

(14) $$L=S_{box}^{}\left(b_{i}^{gt},{\hat{b}}_{i},{\hat{b}}_{i}^{T},{\hat{c}}_{i}^{T}\right)+S_{cls}^{}\left(p_{i}^{gt},{\hat{p}}_{i},{\hat{p}}_{i}^{T},{\hat{c}}_{i}^{T}\right)+S_{cof}^{}\left(c_{i}^{gt},{\hat{c}}_{i},{\hat{c}}_{i}^{T}\right)$$

## 3. Experiments

### 3.1. Dataset

#### 3.1.1. Deep-Sea Biological Dataset

**Figure 6:**
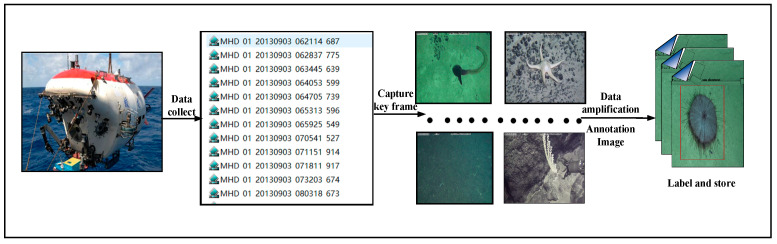
Flow chart of dataset creation.

The dataset in this paper comes from video data taken by a Jiaolong manned submersible on the seabed, and the dataset is constructed according to the process shown in Figure 6. Firstly, key frame images from the video are intercepted, then the images are simply expanded using simple data augmentation such as flipping and rotating, and the images are labeled using the labeling tool to obtain 6144 images and the corresponding labels.

#### 3.1.2. URPC Dataset

**Figure 7:**
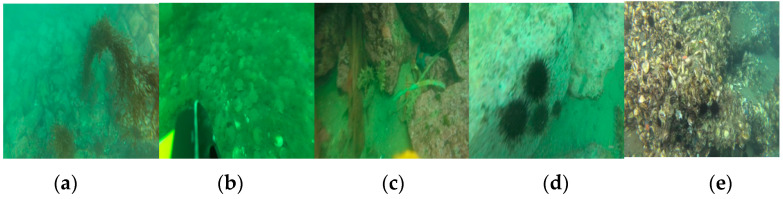
The URPC dataset: (**a**) water plants; (**b**) scallops; (**c**) sea cucumbers; (**d**) sea urchins; (**e**) starfish.

This paper selects the URPC dataset of the Killer Whale open-source project of Pengcheng Laboratory as the source domain dataset during transfer learning. The dataset is curated from the URPC dataset over the years and contains images of multiple scenes, including images taken using underwater robots and divers carrying cameras in shallow seas. The dataset contains a total of 8200 multi-scene images of five categories of underwater biological targets, including sea cucumbers, sea urchins, scallops, starfish, and water plants. Some of these images are shown in Figure 7.

### 3.2. Experimental Environment and Parameter Configuration

The parameters of the experimental environment are shown in Table 1. The optimizer used is stochastic gradient descent, and the learning rate is dynamically adjusted using the cosine annealing algorithm. The batch size is 8 for 100 epochs. The images are all 640 × 640 pixels in size.

### 3.3. Performance Evaluation Metrics for Algorithms

This paper evaluates the lightweights of the model from two aspects: the number of parameters (Param) and the floating point operations (FLOPs), and assesses the detection accuracy of the model using mAP_0.5_ and mAP_0.5:0.95_. Furthermore, the algorithm’s detection speed is evaluated using frames per second (FPS). mAP_0.5_ represents the area under the precision–recall curve when the IOU threshold is set to 0.5. Similarly, mAP_0.5:0.95_ is the weighted average of the areas under the precision–recall curves when the IOU threshold is set from 0.5 to 0.95 with a step size of 0.05. The calculation formulas for precision and recall are as follows:

Precision = *TP*/(*TP* + *FP*) (15)

Recall = *TP*/(*TP* + *FN*) (16)

where *TP* is true positive, *FP* is false positive, and *FN* is false negative.

### 3.4. Experimental Results and Analysis 

#### 3.4.1. Experimental Results Obtained with Lightweight YOLOv5n

**Figure 8:**
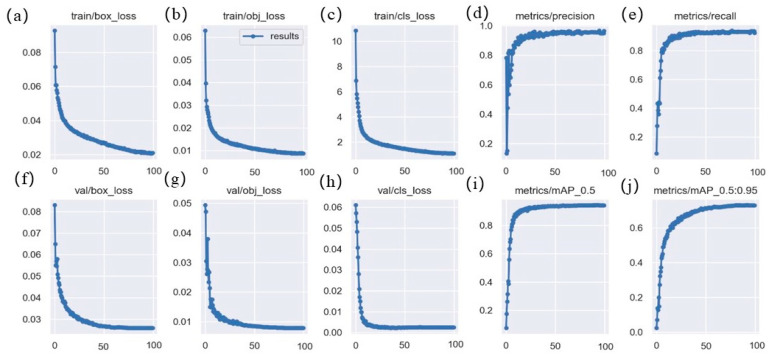
Loss curves of the proposed model on deep-sea biological datasets: (**a**) bounding box regression loss on the training set; (**b**) confidence loss on the training set; (**c**) classification loss on the training set; (**d**) precision on the validation set; (**e**) recall on the validation set; (**f**) bounding box regression loss on the validation set; (**g**)confidence loss on the validation set; (**h**) classification loss on the validation set; (**i**) mAP_0.5_ on the validation set; (**j**) mAP_0.5:0.95_ on the validation set.

The loss of the proposed model on the training dataset, the loss on the validation set, and the change of precision, recall, and mAP are shown in Figure 8, where a, b, and c represent the change curves of the bounding box regression loss, confidence loss, and class loss of the model, and f, g, and h are the change curves of the loss on the corresponding validation set, respectively. From these curves, we can see that as the amount of training increases, the various losses of the model also decrease. This shows that the proposed model has a good fitting effect and high stability, where d, e, i, j are the change curves of various accuracy evaluation indexes of the model on the validation set, and it can be seen from these curves that the detection accuracy of the model increases with the increase of training times. In conclusion, the proposed model has strong fitting ability, good stability, and high accuracy for deep-sea biological detection tasks.

#### 3.4.2. Comparison Experiments with Other Algorithms

To validate the detection performance of the proposed model, we compare the proposed model with the current popular object detection models on the deep-sea biological dataset.

The comparative experimental results are shown in Table 2. Specifically, mAP_0.5_ and mAP_0.5:0.95_ are only slightly lower than YOLOv3 but far superior to YOLOv3 in terms of detection speed and model complexity. In terms of the number of parameters and the amount of computation, the number of parameters is 0.9 M, and the amount of computation is 2.0 GFLOPs, which is the lowest among all models. In terms of FPS, this method is only lower than the fastest YOLOv5n, which can also achieve real-time detection. In addition, we also choose YOLOv8s algorithm, which performs better on public datasets, for comparative experiments. However, from the algorithm performance evaluation metrics shown in Table 2, it can be seen that the YOLOv5-based algorithm has a better balance between performance and accuracy compared with version 8s on the deep-sea biological detection task. This also justifies our choice of YOLOv5n as the base version to implement deep-sea biological detection. In summary, compared with the current popular target detection models, the proposed model has better detection accuracy and lightweight characteristics and achieves a better balance between detection accuracy and lightweight. It is more suitable for exploration missions in deep-sea environments.

#### 3.4.3. Ablation Experiments

**Figure 9:**
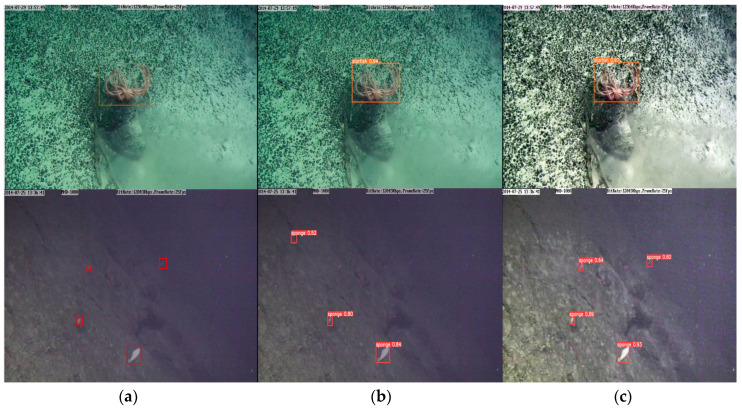
Visualization of detection results: (**a**) labels; (**b**) detection results of Model 2; (**c**) detection results of Model 3.

This section verifies the effectiveness of each submodule and method in deep-sea biological detection tasks. As seen in Table 3, the mAP_0.5_ and mAP_0.5:0.95_ of the original model on the deep-sea biological dataset are 93.6% and 72.9%, and the amount of calculation and parameter is 4.2 GFLOP_S_ and 1.8 M. After introducing GS-Bottleneck into the model, the model’s parameters and computational quantities are reduced by about 50% and 52%. But the detection accuracy is reduced by 0.8% and 1.5% for mAP_0.5_ and mAP_0.5:0.95_. This indicates that the Ghost module and the simAM can guarantee a sure detection accuracy of the model while significantly reducing the model complexity. After introducing image enhancement methods based on global and local contrast fusion, the mAP_0.5_ and mAP_0.5:0.95_ are improved by 1% and 3.3%. Aiming at the serious color cast and low contrast of deep-sea images, two sets of contrast experiments were carried out. The visual inspection results of Model 1 and Model 2 are shown in Figure 9, where Model 2 is based on Model 1 with the image enhancement method we designed. The visual inspection results and the results in Table 3 show that the enhancement method can better correct the color of the image, improve the contrast of the image, increase the confidence of the model detection, reduce the occurrence of missed detection, and then improve the detection accuracy of the model for the degraded image. In brief, the designed image enhancement method can enhance image contrast, correct image color, and improve detection performance. After incorporating the transfer learning combined with knowledge distillation, the model shows a noticeable enhancement in performance. Specifically, it achieves a 1% increase in mAP_0.5_ and a 2% improvement in mAP_0.5:0.95_. This indicates that the strategy can be useful in helping the model to learn similar characteristics to those of deep-sea organisms in shallow-sea organisms and improve the generalization ability through knowledge transfer of the teacher model, which in turn reduces the dependence of the model on the amount of data and improves the detection accuracy of the model. 

**Figure 10:**
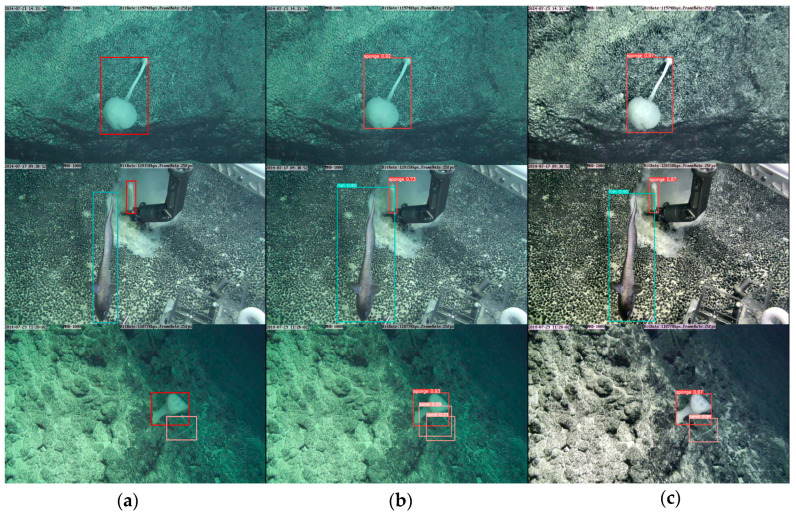
Visualization of detection results: (**a**) labels; (**b**) detection results of YOLOv5n; (**c**) detection results of the proposed method.

Finally, visualizing the detection of the modified model and the unmodified model on the deep-sea biological dataset is shown in Figure 10, where it can be seen that the unmodified model has false detections before the detection of deep-sea organisms. In contrast, the modified model has no false detections. At the same time, the confidence of the improved model in detecting each deep-sea organism is relatively improved. The experimental results demonstrate that each submethod and module proposed in this paper is effective, and the proposed model has higher detection accuracy and lower model complexity than the original model.

## 4. Conclusions

In the real deep-sea environment, aiming at the problem of low detection accuracy caused by poor image quality and insufficient sample size and the problem of high model complexity, we proposed a lightweight YOLOv5n for deep-sea biological detection. The image enhancement method based on local and global contrast fusion can achieve color correction and contrast enhancement for poor-quality deep-sea biological images so as to improve detection accuracy. The GS-Bottleneck module can enable a lightweight model and ensure detection accuracy. Through the transfer learning strategy combined with knowledge distillation, similar characteristics of shallow-sea organisms are transferred to the model, and the generalization ability of the model is further improved by using the knowledge distillation, which can alleviate the impact of insufficient data volume on the detection accuracy. Experimental results demonstrate that our proposed method achieves a notable improvement of 1.2% in mAP_0.5_ and 3.8% in mAP_0.5:0.9_. Remarkably, this is accomplished with a minimal number of parameters (0.9 M) and computational volume (2.0 GFLOPs), making it highly suitable for deployment in deep-sea detection devices for high-precision real-time detection in complex deep-sea imaging environments.

Our method achieved good detection results, but there is still a drawback. Although the inference speed of the model can meet the requirements of real-time detection, it decreased compared with the preimprovement, due to the use of image enhancement methods. Therefore, in future work, we will improve the model to have a faster inference speed. In addition, there is still room for improvement in the fusion part of our image enhancement method, so this method will be further improved in the future to further improve the accuracy of the model for the classification and localization of deep-sea organisms.

## Figures and Tables

**Table 1:** Experimental environment.

Parameters	Configuration
Operating system	Windows 10
GPU	NIVIDIA GeForce RTX3060(12G)
CPU	Intel(R) Core(TM) i5-12490
Acceleration environment	CUDA 11.1
Training framework	Pytorch 1.13.0
Development platform	PyCharm 2023.2.1

**Table 2:** Comparative experimental results of different algorithms.

Model	mAP_0.5_/%	mAP_0.5:0.95_/%	FLOPs/G	Param/M	FPS(CPU)
Faster RCNN	67.9	44.4	370.2	137.1	<1
SSD	78.9	43.1	62.8	26.3	3
YOLOv8s	94.1	76.6	28.8	11.2	9
YOLOv5n	93.6	72.9	4.2	1.8	24
YOLOv3	94.9	79.1	154.7	61.5	5
YOLOv3-Tiny	93.9	74.5	12.9	8.7	11
YOLOv7-Tiny	91.1	67.6	13.1	6.0	10
Proposed method	94.8	76.7	2.0	0.90	12

**Table 3:** Results of ablation experiments.

Model	GS-Bottleneck	GLCF	TLKD	mAP_0.5_/%	mAP_0.5:0.95_/%	FLOPs/G	Param/M
1	×	×	×	93.6	72.9	4.2	1.8
2	√	×	×	92.8	71.4	2.0	0.9
3	√	√	×	93.8	74.7	2.0	0.9
4	√	√	√	94.8	76.7	2.0	0.9

## Data Availability

Not applicable.

## Abbreviations

The following abbreviations are used in this paper:

YOLOv5n	You Only Look Once Version 5n
YOLOv5m	You Only Look Once Version 5m
CLAHE	Contrast-limited adaptive histogram equalization
PCA	Principal component analysis
R-CNN	Region-based convolutional neural networks
Fast R-CNN	Fast region-based convolutional neural networks
MSRCR	Multi-scale retinex with color restoration
simAM	Simple, parameter-free attention module
URPC	Underwater Robot Photography Competition
mAP	Mean average precision
IoU	Intersection over union
